# Case–control study and meta-analysis of *SULT1A1* Arg^213^His polymorphism for gene, ethnicity and environment interaction for cancer risk

**DOI:** 10.1038/sj.bjc.6604683

**Published:** 2008-10-14

**Authors:** A Kotnis, S Kannan, R Sarin, R Mulherkar

**Affiliations:** 1 Genetic Engineering Unit, Advanced Centre for Treatment, Research & Education in Cancer (ACTREC), Tata Memorial Centre, Navi Mumbai, India; 2 Epidemiology and Clinical Trial Unit, ACTREC, Navi Mumbai, India; 3 Cancer Genetics Unit, ACTREC, Tata Memorial Centre, Navi Mumbai, India; 4 Genetic Engineering ACTREC, Tata Memorial Centre, Navi Mumbai, India

**Keywords:** multiple primary neoplasm, SULT1A1, meta-analysis, single nucleotide polymorphism, tobacco related cancer, upper aerodigestive tract cancer

## Abstract

Cytosolic sulphotransferase SULT1A1 plays a dual role in the activation of some carcinogens and inactivation of others. A functional polymorphism leading to Arg^213^His substitution (SULT1A1*2) affects its catalytic activity and thermostability. To study the association of SULT1A1*2 polymorphism with tobacco-related cancers (TRCs), a case–control study comprising 132 patients with multiple primary neoplasm (MPN) involving TRC and 198 cancer-free controls was carried out. One hundred and thirteen MPN patients had at least one cancer in upper aerodigestive tract including lung (UADT-MPN). SULT1A1*2 showed significant risk association with UADT-MPN (odds ratio (OR)=5.50, 95% confidence interval (CI): 1.09, 27.7). Meta-analysis was conducted combining the data with 34 published studies that included 11 962 cancer cases and 14 673 controls in diverse cancers. The SULT1A1*2 revealed contrasting risk association for UADT cancers (OR=1.62, 95% CI: 1.12, 2.34) and genitourinary cancers (OR=0.73, 95% CI: 0.58, 0.92). Furthermore, although SULT1A1*2 conferred significant increased risk of breast cancer to Asian women (OR=1.91, 95% CI: 1.08, 3.40), it did not confer increased risk to Caucasian women (OR=0.92, 95% CI: 0.71, 1.18). Thus risk for different cancers in distinct ethnic groups could be modulated by interaction between genetic variants and different endogenous and exogenous carcinogens.

Tobacco-related cancer (TRC) accounts for almost half the global burden of cancer and arises from a complex gene–environment interaction. Traditionally, only lung, oesophageal, and head and neck cancers were described as TRCs. However, based on several studies, the International Association of Research in Cancer (IARC) has broadened the definition of TRC to include carcinoma of the cervix, bladder, stomach, kidney, pancreas, liver and myeloid leukaemia (Doll, 1999; IARC Working Group on the Evaluation of Carcinogenic Risks to Humans, 2004; Hung *et al*, 2005). Although prolonged exposure to tobacco with or without other carcinogens plays a central role in the genesis of these cancers, various host genetic factors could significantly modulate the risk of developing TRC.

Several genetic alterations in the genes coding for xenobiotic-metabolising enzymes (XMEs), DNA repair, cell cycle regulation and apoptotic pathway confer a low-penetrance genetic susceptibility to tobacco carcinogens (Kotnis *et al*, 2005). There is a large body of evidence, including meta-analyses to support the association of various isoforms of glutathione-*S* transferases (GSTs), cytochrome *P*450 and *N*-acetyl transferase with tobacco carcinogenesis. Although sulphotransferase (SULT) enzymes could play an equally important role in detoxifying tobacco carcinogens, there are very few, mostly inconclusive, studies examining the association of genetic alteration in the genes coding for this super family of multifunctional enzymes with TRC (Seth *et al*, 2000; Hung *et al*, 2004; Sellers *et al*, 2005; Dandara *et al*, 2006).

Sulphotransferase enzymes catalyse sulphation by transferring sulphonate (sulphuryl) group from cofactor 3′-phosphoadenosine 5′-phosphosulphate to a nucleophilic acceptor substrate to form either a sulphate ester or a sulphamate. These sulphate conjugates are more polar and less reactive than the parent compound and facilitate their excretion (Glatt *et al*, 2001). However, some sulpho conjugates are strong electrophiles and may covalently bind to DNA and proteins (Glatt *et al*, 2001).

*SULT1A1* gene is one of the most important and well-studied members of the SULT family and is abundant in a wide variety of tissues. SULT1A1 plays a major role in biotransformation of numerous substrates including several carcinogens, neurotransmitters, steroid hormones and drugs (Raftogianis *et al*, 1999; Hildebrandt *et al*, 2007). Although sulphation is an important property of SULT1A1 in the inactivation of carcinogens, it also plays an important role in toxification of dietary and environmental mutagens (Glatt *et al*, 2001; Al-Buheissi *et al*, 2006). Of the various polymorphisms in *SULT1A1*, the Arg → His polymorphism at position 213, in exon 7 (SULT1A1*2), has a twofold lower catalytic activity and thermo stability than its high-activity Arg^213^ counterpart as demonstrated in platelet cytosol (Raftogianis *et al*, 1997; Nowell *et al*, 2000; Nagar *et al*, 2006). Although several reports show risk association of the *SULT1A1* variant with different cancers (Sun *et al*, 2005; Dandara *et al*, 2006; Pachouri *et al*, 2006; Fan *et al*, 2007; Lilla *et al*, 2007; Bardakci *et al*, 2008), others show either no effect (Sellers *et al*, 2005) or a protective effect (Cheng *et al*, 2005).

In this case–control study, we have examined the association of *SULT1A1* Arg^213^His polymorphism with tobacco carcinogenesis using a unique group of individuals with multiple tobacco-related primary cancers and have used a meta-analysis approach to confirm our findings. Considering that His^213^ variant of SULT1A1 has a lower activity than Arg^213^ variant (Raftogianis *et al*, 1997; Nagar *et al*, 2006), we hypothesise that if tobacco carcinogenesis is significantly modulated by the Arg^213^His polymorphism, it would demonstrate a significant association with TRC and this association may be stronger in individuals with multiple primary TRC.

## Materials and methods

### Study subjects

A registry of patients with multiple primary cancers or familial cancers was established at the Tata Memorial Hospital, Mumbai, in 1996 by one of the authors (RS). From this registry, 132 consecutive multiple primary neoplasm (MPN) patients where one or both the primaries were tobacco related, and their genomic DNA and consent were available, were taken up for this study. Histological or cytological confirmation of each primary cancer was available and each of the cancers was classified as TRC or non-TRC as per the IARC criteria (IARC Working Group on the Evaluation of Carcinogenic Risks to Humans, 2004). There was no restriction for age at diagnosis, gender or carcinogen exposure. For defining two cancers as distinct multiple primaries, modified Hong's criteria (Hong *et al*, 1990) was used, which states that – (a) there is >2 cm of normal intervening mucosa between two primaries in head and neck region; (b) lung as second primary if present, should be of different histology, or be solitary and with characteristic radiology of lung cancer; and (c) there is no evidence of haematogenous spread. Bilateral cancers in paired organs such as breast, ovaries or kidneys were not classified as MPN.

Majority of the MPN cases in the registry hailed from the western and northern parts of India. The cancer-free controls (*n*=198) were volunteers who consented to donate blood or buccal washes for the study. The controls were also from the same region and were free of any cancer or pre-cancerous condition. They were either visiting our hospital in the Preventive Oncology Department for cancer screening (*n*=124) or visiting government dental college for various non-malignant, dental ailments (*n*=68). A few were healthy, ethnically matched workers from Mumbai (*n*=6). Detailed questionnaire including ethnicity and lifetime history of tobacco and alcohol use was obtained from all cases and controls. A majority of them were tobacco users. Family history of cancer was obtained for all MPN cases and from majority of the cancer-free controls. After obtaining informed consent, 3–6 ml of peripheral blood was collected from each subject. Exfoliated buccal cells (mouthwash samples) were collected in sterile phosphate-buffered saline from control individuals who were reluctant to give blood (*n*=68). The study was approved by our Hospital Ethics Committee.

### DNA extraction and genotyping

Genomic DNA was extracted from peripheral blood/mouthwash samples using phenol chloroform method standardised in our laboratory (Koppikar and Mulherkar, 2006). PCR for *SULT1A1* genotyping followed by RFLP using *Hae*II restriction enzyme was carried out as described by Wang *et al* (2002). The authenticity of the PCR products was confirmed by sequencing at least five PCR products at random on an automated DNA sequencer (ABI Prism 3100 Avant) using the Big Dye terminator kit (ABI Prism, Foster City, CA, USA) as per the manufacturer's instructions.

### Identification and analysis of studies for meta-analysis

PUBMED searches were conducted to identify studies on SULT using the search words ‘SULT1A1, SULT *AND* polymorphism’ and ‘SULT *AND* cancer’. The inclusion criteria were case–control studies examining associations of *SULT1A1* Arg^213^His polymorphism either alone or in combination with other genes, published until July 2007. For every study, publication date, country of origin, demographics, genotyping methodology, ethnicity, source and genotype frequency of study subjects were reviewed. In case of missing information, the authors were contacted and requested to provide the data. One study was excluded as all the required information could not be obtained (Peng *et al*, 2003). Genotyping studies on only cancer cases or exclusively on healthy subjects were excluded as comparison of cancer patients with matched controls was a prerequisite for studying association of a particular genotype with cancer risk (Nowell *et al*, 2002b, 2005; Magagnotti *et al*, 2003; Sparks *et al*, 2004; Shatalova *et al*, 2005; Grabinski *et al*, 2006).

### Statistical analysis

The risk (odds ratio, OR) was estimated by comparison of the variant ^213^His genotype *vs* the wild-type ^213^Arg allele using dominant model ((Arg/His+His/His) *vs* Arg/Arg), recessive model (His/His *vs* (Arg/His+Arg/Arg)) as well as the extreme model (His/His *vs* Arg/Arg). The risk was adjusted for age and habit using unconditional logistic regression analysis using SPSS v14.0. Hardy–Weinberg equilibrium in the controls was evaluated for each study using *χ*^2^ test. For each genetic contrast, the between-study heterogeneity was estimated across all eligible comparisons using Q statistics. Funnel plots and Egger’s test were used to assess potential publication bias, which results from non-publication of small studies with negative results (Egger *et al*, 1997). This test detects funnel plot asymmetry by determining whether the intercept deviates significantly from zero in a regression of the standardised effect estimates against their precision. Influence analysis was also carried to assess whether summary OR was driven by any one study in the recessive model of meta-analysis (Sterne *et al*, 2002). Stratification by ethnicity (Asian, Caucasian and Others), total study size of cases and controls (up to 500 or more), Hardy–Weinberg equilibrium (yes/no), primary site (upper aerodigestive tract (UADT) and lung, breast, colorectal, genitourinary and other sites), source of control (population/hospital) and carcinogen exposure studied (yes/no) were pre-specified as characteristics for the assessment of heterogeneity. Meta-analysis was carried out using Review Manager Version 4.2 (Cochrane Collaboration) and STATA software for meta-regression analysis. *P*-values were two sided.

## Results

In this case–control study, we have examined the association of *SULT1A1* Arg^213^His (SULT1A1*2) polymorphism in 132 patients with tobacco-related multiple primary cancers and 198 cancer-free controls. For selection of MPN patients, stringent modified Hong's criteria were used to minimise the possibility of misclassifying metastasis, recurrences, skip lesions and radiation-induced cancers, as second primaries. Radiation therapy was used in the management of first primary in 60% of patients but none of the second primaries were classified as radiation-associated sarcomas (Huber *et al*, 2007) or meningiomas (King *et al*, 2007).

Using the IARC definition of TRC (IARC Working Group on the Evaluation of Carcinogenic Risks to Humans, 2004), these 132 MPN patients with at least one TRC primary were further subclassified as those with at least one primary in the UADT (UADT-MPN, *n*=113, Table 1) and those having none of the primaries in UADT (*n*=19). Majority of the patients (*n*=74) had both the primaries within the UADT region. The characteristics of these 113 patients with at least one primary in UADT and the healthy controls are shown in Table 1. Of the 113 patients with UADT-MPN, 96 (85%) reported tobacco use and majority (68%) had tobacco-chewing habit (Supplementary Table s1). The most common form of tobacco was chewing of tobacco quid with lime or with betel leaf or application of roasted tobacco (masheri) over gums. Quanta and duration of tobacco chewing were not available for all the participants and hence were not included in the analysis.

The genotype distribution of SULT1A1*2 as His/His (homozygous variant), Arg/His (heterozygous) and Arg/Arg (homozygous wild type) was compared in cases and controls using dominant, recessive and extreme models. These models were based on the biological plausibility that His^213^ variant allele is risk conferring compared with Arg^213^ allele. Thus His/His^213^ genotype was considered risk conferring whereas Arg/Arg^213^ would confer protection. However, activity of the SULT1A1 allozymes in platelets from heterozygous (Arg/His) individuals has been reported to be only slightly lower than that from the Arg/Arg individuals but much higher than that from His/His individuals (Raftogianis *et al*, 1997; Nowell *et al*, 2000). Hence recessive model (Arg/Arg+Arg/His *vs* His/His) was considered in the study.

The risk association of SULT1A1*2 was evaluated in 113 MPN patients with at least one UADT TRC. The remaining 19 patients with both TRCs outside UADT were analysed as a separate group as well as a combined TRC group (*n*=132) (Table 2). The results of the TRC outside UADT group (*n*=19) and the combined group (*n*=132) are not included in the meta-analysis due to small sample size (*n*=19) with very diverse TRCs (cervix, bladder and stomach and so on). After adjusting for age and tobacco use, a significant risk association of SULT1A1*2 with UADT TRC was seen in dominant, recessive as well as extreme models. In all three models, there was a significant increased risk associated with His^213^ genotype (Table 2).

**Figure 1:**
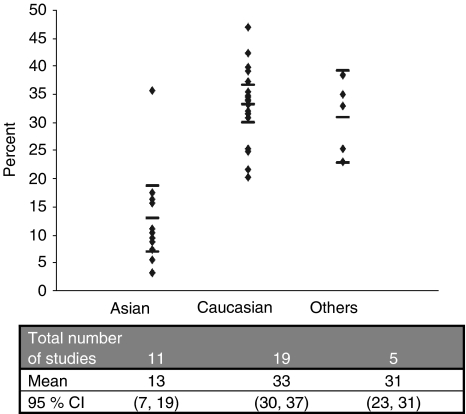
Allele frequencies from the meta-analysis studies in control groups of different ethnicities.

To compare these observations with the published studies, a meta-analysis of studies evaluating association of *SULT1A1* Arg^213^His with different cancers was performed. From the Medline search using the search terms described earlier, we identified 34 case–control studies for *SULT1A1* Arg^213^His polymorphism (Seth *et al*, 2000; Steiner *et al*, 2000; Bamber *et al*, 2001; Zheng *et al*, 2001, 2003; Ozawa *et al*, 2002; Wang *et al*, 2002; Nowell *et al*, 2002a, 2004; Tang *et al*, 2003; Wu *et al*, 2003; Chacko *et al*, 2004; Hung *et al*, 2004; Langsenlehner *et al*, 2004; Liang *et al*, 2004; Tiemersma *et al*, 2004; Tsukino *et al*, 2004; Boccia *et al*, 2005, 2006; Cheng *et al*, 2005; Choi *et al*, 2005; Han *et al*, 2005; Jerevall *et al*, 2005; Le Marchand *et al*, 2005; Lilla *et al*, 2005; Moreno *et al*, 2005; Pereira *et al*, 2005; Sellers *et al*, 2005; Sun *et al*, 2005; Yang *et al*, 2005; Dandara *et al*, 2006; Kellen *et al*, 2006; Mikhailova *et al*, 2006; Pachouri *et al*, 2006). Including the 113 patients with UADT-MPN from this study, there were 11 962 cancer cases and 14 673 cancer-free controls. To specifically examine the risk association of His^213^ with different types of cancers including UADT TRC, which is biologically more plausible, the studies included in meta-analysis were categorised according to the site of primary cancer as UADT TRC, genitourinary, breast, colorectal and other cancer sites. All the studies were further analysed with respect to genotypes, source of controls, ethnicity and carcinogen exposure. The study characteristics (Supplementary Table s2) showed that 13 studies had accrued controls from general population whereas 20 studies had hospital-based controls and one had mixed source of controls. In 23 studies, the distribution of genotypes in controls was consistent with Hardy–Weinberg equilibrium (Supplementary Table s3). It was noteworthy that the His^213^ allele occurred at a significantly lower frequency amongst Asians (13%; 95% confidence interval (CI): 7–19) as compared with Caucasians (33%; 95% CI: 30–37) and other ethnic groups (31%; 95% CI: 23–31) (Figure 1). However, it is possible that the variation is even larger between different Asian populations.

**Figure 2:**
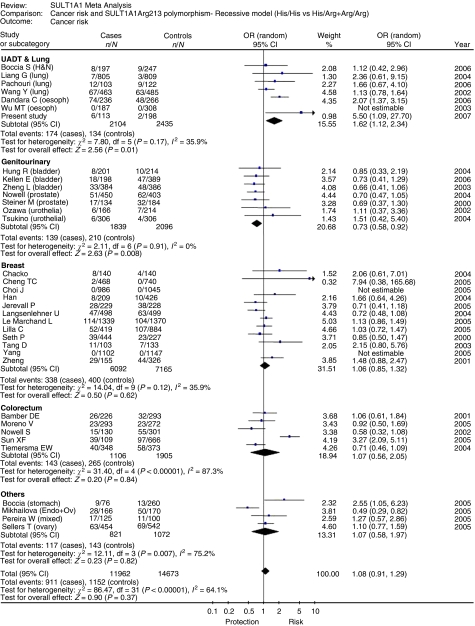
Meta-analysis recessive model.

All the studies were analysed using the three models, namely recessive (Figure 2), which was biologically more plausible (Raftogianis *et al*, 1997), as well as dominant and extreme models (Supplementary Figure s1, s2 and Supplementary Table s3). All the three models showed a high degree of statistical heterogeneity among the 35 studies, including this study. Meta-regression analysis was performed to investigate the source for statistical heterogeneity (Supplementary Table s3). No obvious source of heterogeneity was identified except for ethnicity in the dominant model.

Symmetrical Funnel plot suggested the absence of publication bias for all the three models (Egger's test *P*-value >0.05; Supplementary Figure s3). Influence analysis was carried out to study the effect of individual studies in the meta-analysis on the overall outcome (Supplementary Figure s4). None of the studies affected the outcome of the meta-analysis significantly. When different ethnic groups were analysed separately irrespective of cancer site, the Asians showed a significant increased risk (OR=1.84, 95% CI: 1.20, 2.83) as compared with Caucasians (OR=1.03, 95% CI: 0.82, 1.29) (Supplementary Table s3) in the recessive model.

The effect of ethnicity for specific cancer sites could be examined separately only for breast cancer where ethnicity was reported in sufficient number of studies (*n*=11) (Supplementary Figure s5). Effect of ethnicity could not be evaluated in other cancer sites due to the small number of studies. Although SULT1A1*2 conferred significant increased risk of breast cancer to Asian women (OR=1.91, 95% CI: 1.08, 3.40), it did not confer increased risk to Caucasian women (OR=0.92, 95% CI: 0.71, 1.18).

Stratified meta-analysis according to cancer site, irrespective of ethnicity or any other factor, showed a 1.46- to 1.62-fold risk for UADT cancer in all the three models, whereas the cancers in the genitourinary site showed a significant protection with an OR of 0.67–0.81 in the three models (Figure 2, Supplementary Figure s1 and s2). Other tumour sites, however, did not show any significant association.

## Discussion

Despite decades of public health programmes, TRCs remain the leading cause of cancer morbidity and mortality worldwide. Epidemiological studies over the past 50 years have clearly established how tobacco contributes to cancer risk not only in the directly exposed and anatomically related regions of the upper aerodigestive tract and lung but also in distant organs such as cervix, bladder, kidney, pancreas and so on (IARC Working Group on the Evaluation of Carcinogenic Risks to Humans, 2004). Tobacco is implicated as the single most important environmental factor for several TRCs (lung, head and neck, oesophagus, bladder, kidney and pancreas). It also confers significant risk for cancers even where viral or bacterial oncogenesis plays a predominant role (e.g., cervix, stomach, liver and nasopharynx). Weak genetic susceptibility in tobacco-exposed population is conferred by a large number of low-penetrance genes. However, there is paucity of systematic studies of all the important genes that may predispose to tobacco carcinogenesis.

The focus of research to elucidate genetic susceptibility to tobacco carcinogenesis has been on phase I and phase II detoxifying enzymes and to a lesser extent on the genes that regulate DNA repair, apoptosis and other relevant pathways. In contrast to the GST super family of enzymes that have been studied extensively for tobacco carcinogenesis (Nakajima *et al*, 1995; Cheng *et al*, 1999; Buch *et al*, 2002; Jhavar *et al*, 2004, 2005), other phase II metabolising enzymes such as the SULT have been less extensively studied (Hung *et al*, 2004; Dandara *et al*, 2006; Pachouri *et al*, 2006). There also has not been any collation of published data or a meta-analysis of case–control studies evaluating SULT enzyme in cancers.

Yasuda *et al* (2007) have reported that of the 11 known human cytosolic sulphotransferases, SULT1A1 is one of the four major SULT enzymes responsible for sulphation of tobacco carcinogens. The role of SULT1A1 in the biotransformation of tobacco carcinogens and its association with lung cancer has been previously reported (Liang *et al*, 2004). There are reports of SULT1A1*2 association with increased risk for oesophageal cancers (Dandara *et al*, 2006) as well as gastric cancer (Boccia *et al*, 2005) in individuals who consume alcohol and smoke tobacco.

To elucidate the effect of Arg^213^His polymorphism of *SULT1A1* in tobacco users, we have studied a group of patients with multiple primary cancers, where at least one of the primary cancers was a TRC. We have postulated that as opposed to patients with a single primary TRC, those who develop multiple primary cancers are likely to show more pronounced gene–environment interactions (Kotnis *et al*, 2005). Hence, this may be a better clinical model to detect significant association of low-penetrance genes, even in smaller number of patients. In this case–control study, we show a strong association of the Arg^213^His polymorphism of *SULT1A1* with the development of tobacco-related UADT cancers. These findings are further supported by the results of the meta-analysis examining the association of this polymorphism with cancer risk.

The meta-analysis also brings out the markedly lower mean frequency of SULT1A1 His^213^ in the Asian population as compared with the Caucasian population. However, it is possible that the variation is even larger if different Asian populations are taken in the study separately. We (Jhavar *et al*, 2004, 2005) and others from India (Buch *et al*, 2002; Anantharaman *et al*, 2007) have reported a markedly lower frequency of *GSTT1* null genotype in the Indian population as compared with that in the Japanese, Chinese and Korean population (Raimondi *et al*, 2006). Although marked geoethnic variation in the incidence of different cancers is attributed largely to the differences in carcinogenic exposure and diet, marked differences in the population frequency of the risk-conferring genotype of some XMEs could also influence cancer risk.

The results of this meta-analysis are intriguing as they demonstrate opposite effects of *SULT1A1* polymorphism on two distinct anatomical sites of TRCs. Thus in the meta-analysis, in contrast to the seven studies where UADT and lung cancers showed an increased risk association with SULT1A1*2, seven studies on genitourinary cancers showed a protective effect. This could perhaps be explained by the dual role of SULT1A1 in the bioactivation as well as detoxification of carcinogens (Glatt, 2000). Thus, detoxification of exogenous and endogenous carcinogens confers a protective effect for cancer (Glatt *et al*, 2001), whereas bioactivation of promutagens could increase the risk of certain cancers (Zheng *et al*, 2003; Tiemersma *et al*, 2004). The risk association of SULT1A1*2 with cancers of the UADT and lung is expected from its known role in tobacco detoxification (Al-Buheissi *et al*, 2006). Nowell *et al* (2004) has reported that *SULT1A1* could contribute to prostate cancer risk, and the magnitude of the association may depend on ethnicity and meat consumption. It has been reported that the carcinogens are transferred to the kidney and ureter (Meinl *et al*, 2006) although their levels are substantially lower in the kidney than in the liver. However, it is difficult to explain the protective role of SULT1A1*2 His^213^ variants with cancer in the genitourinary cancers. Detailed biochemical studies in different human tissues, especially in the genitourinary *vs* UADT region, might explain the opposing tissue-specific effects of *SULT1A1*.

Another important aspect that has emerged from the meta-analysis is the difference in the risk of breast cancer conferred by SULT1A1*2 variant to Asian women compared with Caucasian women. A similar phenomenon has been reported for *GSTM1* polymorphism. Carlsten *et al* (2008) have reported that although *GSTM1* null status conferred a significantly increased risk of lung cancer to East Asians it did not confer increased risk to Caucasians. Thus, in distinct ethnic groups, risk for different cancers could be modulated by interaction between genetic variants and different endogenous and exogenous carcinogens.

There are several limitations in the present meta-analysis as is often the case. Contribution of possible sources of heterogeneity such as site of cancer, ethnicity, Hardy–Weinberg equilibrium, source of controls, sample size/power and carcinogen exposure were considered. However, meta-regression analysis demonstrated a significant heterogeneity due to ethnicity alone. This was also reflected in the allele frequency where Asians and Caucasians showed a striking difference. Hence, the actual source of heterogeneity could not be investigated due to the complexities of the confounding variables. In addition, meta-analysis in general looks at the crude OR instead of adjusted OR as the adjustment and matching factors differ across the studies. The residual confounders might have influenced our analysis.

This study encourages detailed biochemical investigation on the-tissue specific influence of SULT1A1 Arg^213^His enzyme in metabolism of tobacco carcinogens. This is the first meta-analysis that provides significant and contrasting association of *SULT1A1* Arg^213^His polymorphism on cancer risk in distinct sites of TRCs namely UADT and genitourinary and an increased risk for breast cancer in Asians.

## Figures and Tables

**Table 1:** Demographics of study subjects

**Category**	**UADT TRC^a^ (*n*=113) (%)**	**Cancer-free controls^b^ (*n*=198) (%)**
*Gender*		
Males	74 (65)	129 (65)
Females	39 (35)	69 (35)
		
*Age* c		
Median	50	46
Range	(26–75)	(20–84)
		
*Type of MPN*		
Synchronous^d^	32 (28)	—
Metachronous	79 (70)	—
		
	Oral – 90	—
	Oesophagus – 28	—
	Larynx/hypopharynx – 24	—
Primary cancer sites (226 cancers in 113 cases)	Oropharynx – 24	—
	Lung – 15	—
	Cervix – 15	—
	Others – 30	—
		
*Tobacco habit*		
No habit	14 (12)	14 (7)
Only T	70 (62)	156 (79)
T+A	26 (23)	27 (14)
No information	3 (3)	1 (<1)
		
*SULT1A1 genotypes*		
Arg/Arg	60 (53)	135 (68)
Arg/His	47 (42)	61 (31)
His/His	6 (5)	2 (1)

A=alcohol; MPN=multiple primary neoplasm; T=tobacco; TRC=tobacco-related cancer; UADT=upper aerodigestive tract.

Tobacco-related cancers were as defined by IARC (2004) and included UADT (including nasopharynx), cervix, bladder, stomach, kidney, liver, pancreas and myeloid leukaemia.

a At least one primary in the UADT.

b Controls were enrolled mainly from the Preventive Oncology Department, Tata Memorial Hospital and Government Dental College, Mumbai.

c Age (years) at the diagnosis of the index cancer of the patients or age at accrual for the controls.

d Synchronous – cancers occurring within 6 months of diagnosis of first primary site.

**Table 2:** Analysis of risk association in tobacco-related MPN patients using genetic models

		**Cancer-free controls (*n*=198)**	**TRC outside UADT (*n*=19)**	**At least one in UADT (*n*=113)**	**All TRCs (*n*=132)**
**Category**	**Genotype**	***N***	***n* (OR^a^ (95% CI))**	***n* (OR^a^ (95% CI))**	***n* (OR^a^ (95% CI))**
Dominant	Arg/Arg	135	6 (ref)	60 (ref)	66 (ref)
	His/His,His/Arg	2, 61	0, 13 (7.91 (2.06, 30.39))	6, 47 (1.94 (1.20, 3.14))	6, 60 (2.12 (1.3, 3.44))
Recessive	His/Arg,Arg/Arg	61, 135	13, 6 (ref)	47, 60 (ref)	60, 66 (ref)
	His/His	2	0	6 (6.07 (1.20, 30.66))	6 (7.43 (1.42, 38.820)
Extreme	Arg/Arg	135	6 (ref)	60 (ref)	66 (ref)
	His/His	2	0	6 (7.84 (1.53, 40.15))	6 (8.92 (1.71, 46.66))

MPN=multiple primary neoplasm; TRC=tobacco-related cancer; UADT=upper aerodigestive tract.

a Odds ratio (OR) adjusted for age and tobacco habit; 95% CI – 95% confidence interval.
