# Supplementary material for: Case–control study and meta-analysis of SULT1A1 Arg213His polymorphism for gene, ethnicity and environment interaction for cancer risk
Source: Br J Cancer. 2008 Oct 14;99(8):1340–7. doi: 10.1038/sj.bjc.6604683 (PMC2570530; doi:10.1038/sj.bjc.6604683)
Supplement: Supplementary Information [file 6604683x1.doc]

**Supplementary Fig s1 Meta-Analysis Dominant model**

**
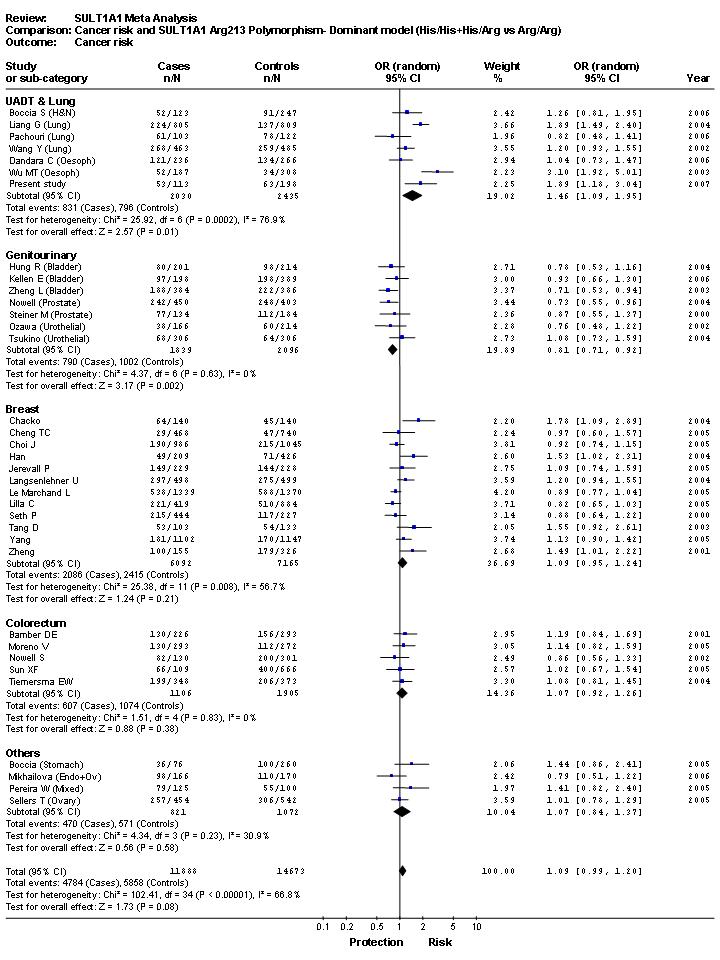
**

**Supplementary Fig s2 – Meta-analysis Extreme Model**


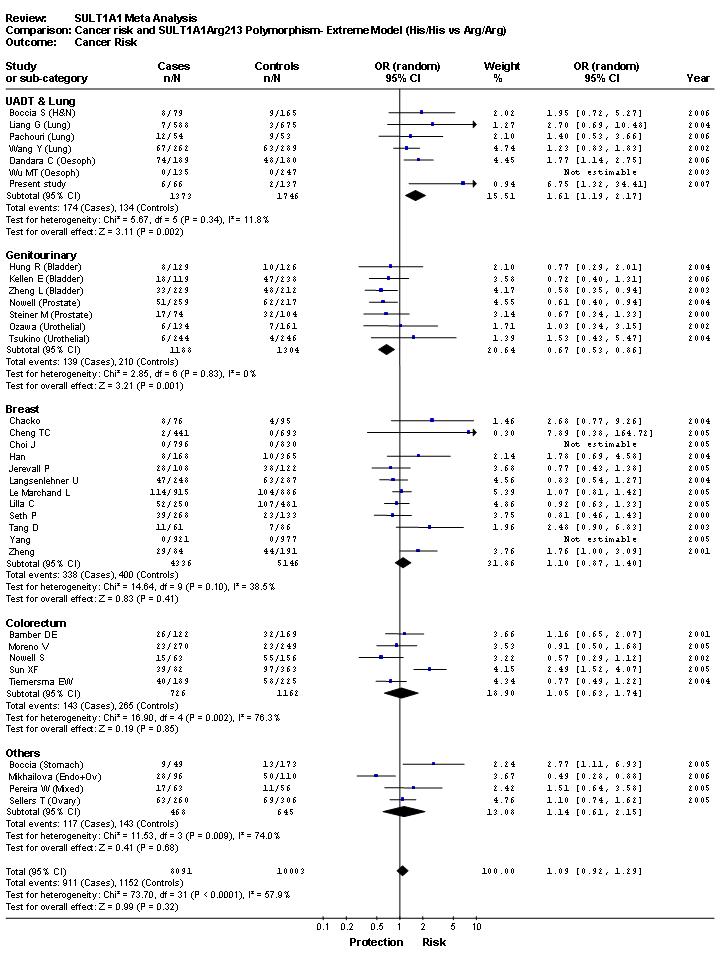


**Supplementary Fig s3 – Funnel Plots
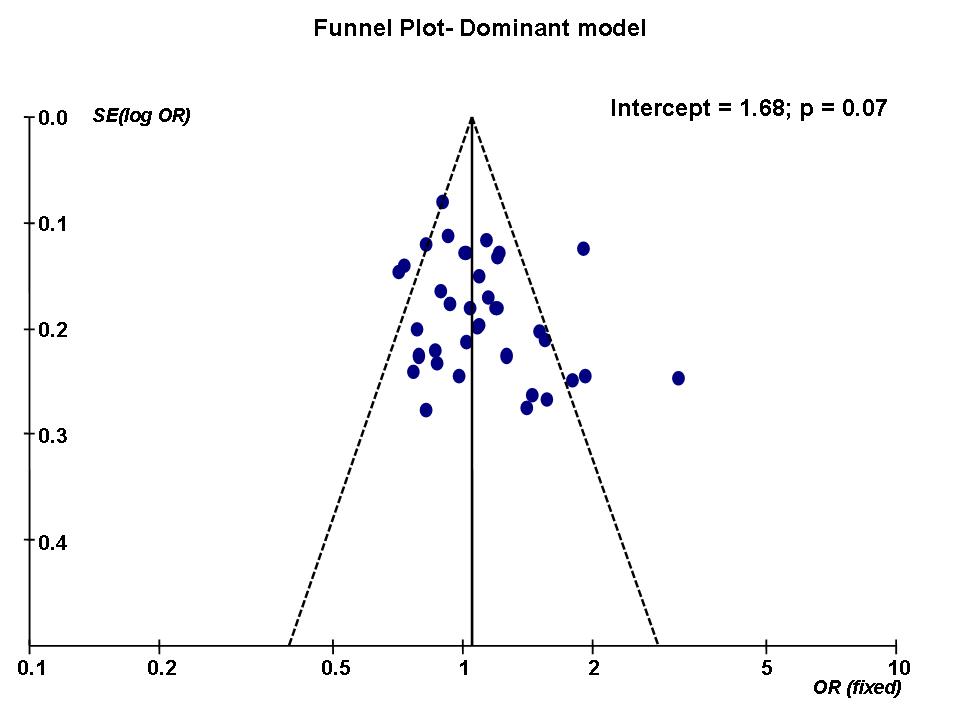
**

*^

**
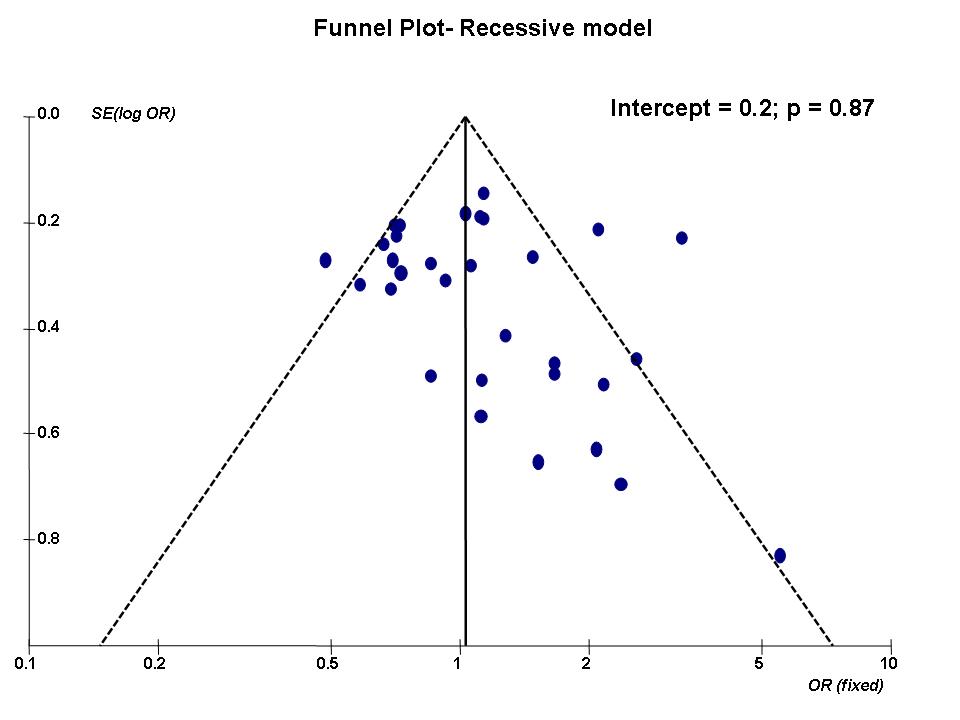
**

*^

**
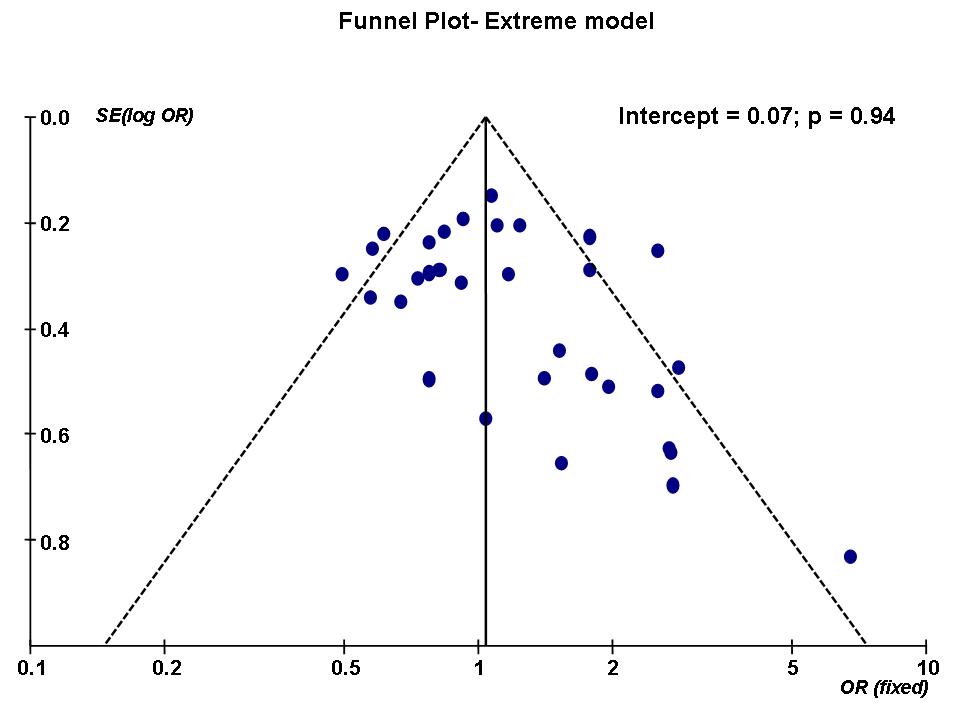
**

*** Four studies (Cheng et al., 2005), (Choi et al., 2005), (Wu et al., 2003) & (Yang et al., 2005) were removed as there is no representation of His/His genotype.**

**Supplementary Fig s4 – Influence Analysis (Recessive model)**

**
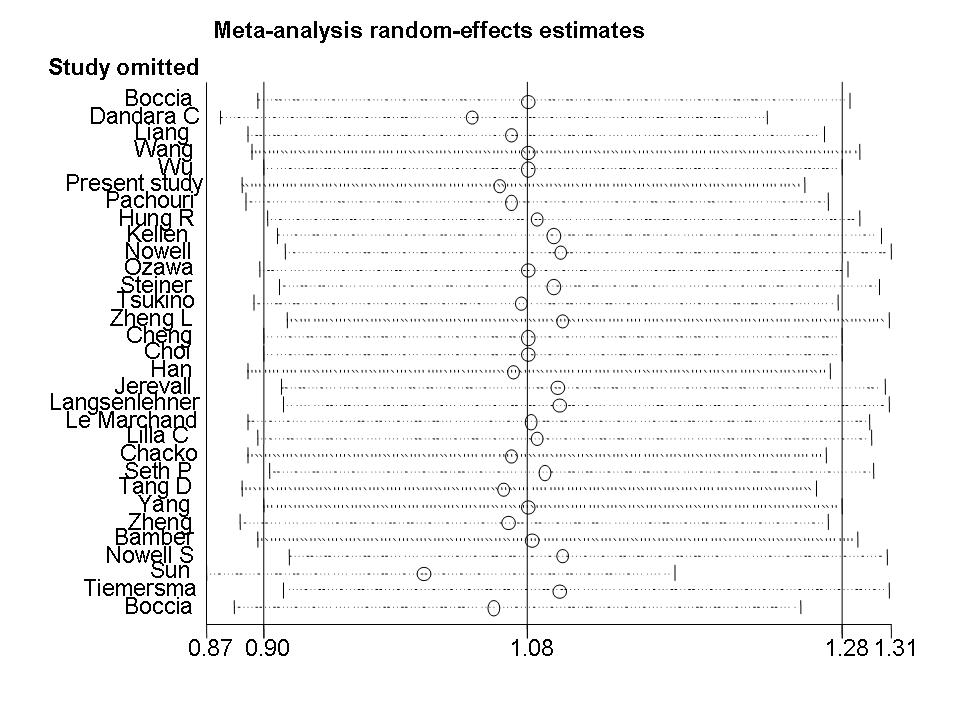
**

# Supplementary Fig s5: Effect of Ethnicity on Breast Cancer – SULT1A1 Meta-analysis by Recessive Model

**
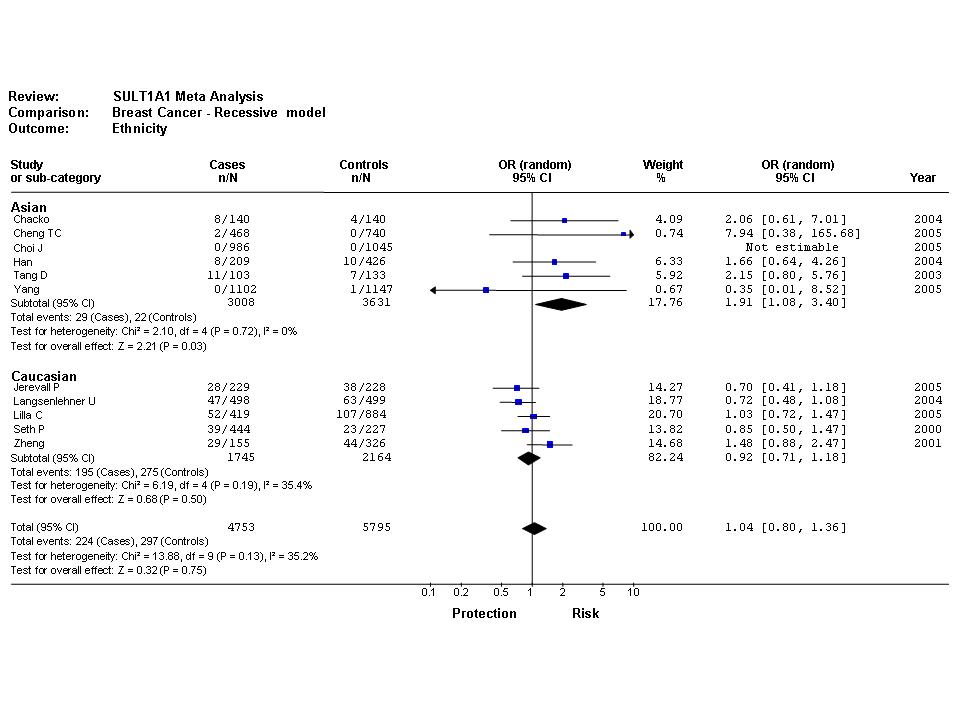
**

**Supplementary Table s1: Pattern of tobacco use in 113 patients and controls**

|  | | Cases | | Controls | |
| --- | --- | --- | --- | --- | --- |
|  |  | n | % | n | % |
| Type of Tobacco use | C | 43 | 38 | 126 | 64 |
|  | S | 8 | 7 | 16 | 8 |
|  | C+A | 4 | 4 | 13 | 7 |
|  | S+A | 11 | 10 | 6 | 3 |
|  | C+S | 19 | 17 | 14 | 7 |
|  | C+S+A | 11 | 10 | 8 | 4 |
|  | Nil | 14 | 12 | 13 | 7 |
|  | Missing | 3 | 3 | 1 | 1 |
|  | A |  |  | 1 |  |
|  | Total | 113 |  | 198 |  |
|  | Chi-square, p | 26.33, 0.00008 | | | |
|  | | | | | |
| T in any or both form | C,S,C+S | 70 | 73 | 156 | 85 |
| T in any or both form with Alcohol | C+A,S+A,C+S+A | 26 | 27 | 27 | 15 |
|  | Chi-square, p | 6.22, 0.013 | | | |
|  | | | | | |

C- Tobacco chewing

S- Tobacco smoking

A- Alcohol

T- Tobacco

**Supplementary Table s2 Characteristics of studies included in meta-analysis**

| **No.** | **Author** | **Year** | **Cancer** | **Source of controls** | **Cases/Controls** | | | **Total**  **Cases/Controls** |
| --- | --- | --- | --- | --- | --- | --- | --- | --- |
|  |  |  |  |  | **(Arg/Arg) (Jackson et al.)** | **Arg/His (HT)** | **His/His (HM)** |  |
| 1 | (Yang et al., 2005) | 2005 | Breast | Population | 921/977 | 181/170 | 0/0 | 1102/1147 |
| 2 | (Boccia et al., 2005) | 2005 | Gastric | Hospital | 40/160 | 27/87 | 9/13 | 76/260 |
| 3 | (Zheng et al., 2003) | 2003 | Bladder | Hospital | 196/164 | 155/174 | 33/48 | 384/386 |
| 4 | (Tsukino et al., 2004) | 2004 | Urothelial | Hospital | 238/242 | 62/60 | 6/4 | 306/306 |
| 5 | (Zheng et al., 2001) | 2001 | Breast | Population | 55/147 | 71/135 | 29/44 | 155/326 |
| 6 | (Seth et al., 2000) | 2000 | Breast | Hospital | 229/110 | 176/94 | 39/23 | 444/227 |
| 7 | (Kellen et al., 2006) | 2006 | Bladder | Population | 101/191 | 79/151 | 18/47 | 198/389 |
| 8 | (Sun et al., 2005) | 2005 | Colorectum | Population | 43/266 | 27/303 | 39/97 | 109/666 |
| 9 | (Mikhailova et al., 2006) | 2006 | Endomet, Ovary | Hospital | 68/60 | 70/60 | 28/50 | 166/170 |
| 10 | (Sellers et al., 2005) | 2005 | Ovary | Hospital | 197/236 | 194/237 | 63/69 | 454/542 |
| 11 | (Dandara et al., 2006) | 2006 | Oesophagus | Population | 115/132 | 47/86 | 74/48 | 236/266 |
| 12 | (Han et al., 2005) | 2005 | Breast | Hospital | 160/355 | 41/61 | 8/10 | 209/426 |
| 13 | (Jerevall et al., 2005) | 2005 | Breast | Population | 80/84 | 121/106 | 28/38 | 229/228 |
| 14 | (Le Marchand et al., 2005) | 2005 | Breast | Population | 801/782 | 424/484 | 114/104 | 1339/1370 |
| 15 | (Pereira et al., 2005) | 2005 | Mixed | Hospital | 46/45 | 62/44 | 17/11 | 125/100 |
| 16 | (Choi et al., 2005) | 2005 | Breast | Mixed | 796/830 | 190/215 | 0/0 | 986/1045 |
| 17 | (Lilla et al., 2005) | 2005 | Breast | Population | 198/374 | 169/403 | 52/107 | 419/884 |
| 18 | (Cheng et al., 2005) | 2005 | Breast | Hospital | 439/693 | 27/47 | 2/0 | 468/740 |
| 19 | (Hung et al., 2004) | 2004 | Bladder | Hospital | 121/116 | 72/88 | 8/10 | 201/214 |
| 20 | (Langsenlehner et al., 2004) | 2004 | Breast | Population | 201/224 | 250/212 | 47/63 | 498/499 |
| 21 | (Moreno et al., 2005) | 2005 | Breast | Hospital | 163/160 | 107/89 | 23/23 | 293/272 |
| 22 | (Nowell et al., 2004) | 2004 | Prostate | Population | 208/155 | 191/186 | 51/62 | 450/403 |
| 23 | (Liang et al., 2004) | 2004 | Lung | Population | 581/672 | 217/134 | 7/3 | 805/809 |
| 24 | (Tiemersma et al., 2004) | 2004 | Colorectum | Hospital | 149/167 | 159/148 | 40/58 | 348/373 |
| 25 | (Wu et al., 2003) | 2003 | Esophageal | Hospital | 135/274 | 52/34 | 0/0 | 187/308 |
| 26 | (Tang et al., 2003) | 2003 | Breast | Hospital | 50/79 | 42/47 | 11/7 | 103/133 |
| 27 | (Ozawa et al., 2002) | 2002 | Urothelial | Hospital | 128/154 | 32/53 | 6/7 | 166/214 |
| 28 | (Nowell et al., 2002a) | 2002 | Colorectum | Population | 48/101 | 67/145 | 15/55 | 130/301 |
| 29 | (Steiner et al., 2000) | 2000 | Prostate | Hospital | 57/72 | 60/80 | 17/32 | 134/184 |
| 30 | (Bamber et al., 2001) | 2001 | Colorectum | Hospital | 96/137 | 104/124 | 26/32 | 226/293 |
| 31 | (Wang et al., 2002) | 2002 | Lung | Population | 195/226 | 201/196 | 67/63 | 463/485 |
| 32 | (Boccia et al., 2006) | 2006 | Head-Neck | Hospital | 71/156 | 44/82 | 8/9 | 123/247 |
| 33 | (Chacko et al., 2004) | 2004 | Breast | Hospital | 76/95 | 56/41 | 8/4 | 140/140 |
| 34 | (Pachouri et al., 2006) | 2006 | Lung | Hospital | 42/44 | 49/69 | 12/9 | 103/122 |

# Supplementary Table s3: Summary Odds Ratio by Genetic Models

| **Category** | **Sub-category** | **No. of studies** | **Dominant** | | **Recessive** | | **Extreme** | |
| --- | --- | --- | --- | --- | --- | --- | --- | --- |
|  |  |  | **I^2^ (%)** | **OR (95% CI)** | **I^2^ (%)** | **OR (95% CI)** | **I^2^ (%)** | **OR (95% CI)** |
| Cancer Site | UADT & Lung | 7 | 77 | 1.46 (1.09, 1.95) | 36 | 1.62 (1.12, 2.34) | 12 | 1.61 (1.19, 2.17) |
|  | Genitourinary | 7 | 0 | 0.81 (0.71, 0.92) | 0 | 0.73 (0.58, 0.92) | 0 | 0.67 (0.53, 0.86) |
|  | Breast | 13 | 57 | 1.09 (0.95, 1.24) | 36 | 1.06 (0.85, 1.32) | 39 | 1.10 (0.87, 1.40) |
|  | Colorectal | 4 | 0 | 1.07 (0.92, 1.24) | 87 | 1.07 (0.56, 2.05) | 76 | 1.05 (0.63, 1.74) |
|  | Others | 4 | 31 | 1.07 (0.84, 1.37) | 75 | 1.07 (0.58, 1.97) | 74 | 1.14 (0.61, 2.15) |
| Meta-regression coefficient | | | | 0.03 | 0.002 | | 0.008 | |
| Ethnicity | Asian | 11 | 79 | 1.31 (1.04,1.66) | 0 | 1.84 (1.20, 2.83) | 0 | 1.90 (1.23, 2.94) |
|  | Caucasian | 19 | 7 | 1.03 (0.95, 1.12) | 73 | 1.03 (0.82, 1.29) | 62 | 0.97 (0.79, 1.19) |
|  | Others | 5 | 66 | 0.92 (0.73, 1.16) | 57 | 0.96 (0.68, 1.34) | 69 | 1.20 (0.78, 1.86) |
| Meta-regression coefficient | | | | -0.22^$^ | -0.25 | | -0.27 | |
| HWE | Yes | 23 | 75 | 1.13 (0.97, 1.31) | 63 | 1.08 (0.86, 1.36) | 62 | 1.13 (0.89, 1.43) |
|  | No | 12 | 23 | 1.02 (0.92, 1.13) | 70 | 1.09 (0.81, 1.47) | 59 | 1.08 (0.83, 1.41) |
| Meta-regression coefficient | | | | -0.03 |  | -0.05 |  | -0.06 |
| Sample Size | <= 500 | 15 | 73 | 1.15 (0.92, 1.44) | 62 | 1.03 (0.72, 1.47) | 60 | 1.22 (0.86, 1.74) |
|  | > 500 | 20 | 66 | 1.03 (0.93, 1.15) | 72 | 1.10 (0.88, 1.38) | 58 | 1.04 (0.86, 1.26) |
| Meta-regression coefficient | | | | -0.07 |  | 0.02 |  | -0.08 |
| Source of controls ^#^ | Hospital | 21 | 73 | 1.17 (0.99, 1.37) | 40 | 1.03 (0.83, 1.28) | 48 | 1.10 (0.87, 1.40) |
|  | Population | 13 | 74 | 1.06 (0.92, 1.24) | 79 | 1.09 (0.91, 1.29) | 70 | 1.08 (0.84, 1.39) |
| Meta-regression coefficient | | | | 0.07 |  | 0.09 |  | 0.06 |
| Carcinogen exposure studied | Yes | 19 | 78 | 1.12 (0.94, 1.32) | 50 | 1.18 (0.94, 1.48) | 54 | 1.21 (0.94, 1.54) |
|  | No | 16 | 34 | 1.02 (0.92, 1.12) | 74 | 0.99 (0.76, 1.30) | 63 | 0.99 (0.78, 1.26) |
| Meta-regression coefficient | | | | -0.05 |  | -0.14 |  | -0.16 |

^$^ P = 0.01

^#^Choi et al had selected controls from mixed population
